# MRI measurements of pancreatic fat content in the general population and their relationship with age, gender, and body mass index

**DOI:** 10.1097/MD.0000000000048133

**Published:** 2026-03-27

**Authors:** Xue Qi, Fang Zhang, Wenjun Yao, Xiaoguang Cheng, Liming Guan

**Affiliations:** a Department of Radiology, Ordos Central Hospital, Ordos, Inner Mongolia, China; b Department of Radiology, The Second Affiliated Hospital of Anhui Medical University, Hefei, Anhui, China; c Department of Radiology, Beijing Jishuitan Hospital, Beijing, China; d Department of Radiology, First Affiliated Hospital of China Medical University, Shenyang, Liaoning, China.

**Keywords:** age, BMI, gender, MR imaging, pancreas, proton density fat fraction

## Abstract

Increased fat deposition in the pancreas is known to be associated with an increased risk of type 2 diabetes mellitus and metabolic syndrome. However, few studies have measured the pancreatic fat content in a normal population or tried to set a threshold for defining nonalcoholic fatty pancreatic disease. Two hundred thirty-seven healthy subjects (107 males and 130 females; age range of 21–79 years; mean age of 50 ± 15 years) underwent 3-point Dixon magnetic resonance imaging to measure the proton density fat fraction (PDFF) in the head, body, and tail of the pancreas. Subjects were divided into 5 age ranges (21–30, 31–40, 41–50, 51–60, and > 60 years old). After testing data for normality, parametric and nonparametric statistical tests were used to study how pancreatic fat content varied with gender, age, and body mass index. Median pancreatic fat content was 3.33% (inter quartile range 1.82–6.16%) and did not vary significantly between the head, body, and tail. Over all ages, men had higher pancreatic PDFF than women (*P* = .010) as well as in each of the 5 age ranges. The trend for pancreatic fat content to increase with age was highly statistically significant (*P* < .001). In men, median PDFF increased from 3.22% in the 21 to 30-year-old group to 6.26% in the > 60 group. In women, the same figures were 1.44% and 4.41%. There was also a statistically significant increase in pancreatic PDFF with body mass index. Pancreatic fat was homogeneous and evenly distributed in the head, body, and tail of the pancreas. Pancreatic PDFF was higher in men, elderly people, and in obesity.

## 1. Introduction

Obesity has become a growing health concern, affecting many people worldwide. There are several increased health risks linked to obesity including metabolic and cardiovascular disorders linked with visceral and ectopic organ fat deposition.^[1–3]^ Pancreatic fat deposition, also known as nonalcoholic fatty pancreatic disease, refers to the accumulation of fat in pancreatic acinar or pancreatic islet cells or the replacement of pancreatic parenchyma by adipose tissue, which is a kind of heterotopic deposition of visceral fat. Previous studies showed that excessive fat deposition in the pancreas may result in a decreased number of pancreatic cells, decreased insulin secretion, and apoptosis of pancreatic islets.^[4,5]^ Nonalcoholic fatty pancreatic disease is associated with at least a 2-fold increased risk of type 2 diabetes mellitus (DM), metabolic syndrome, and its associated hypertension.^[6]^ Moreover, it is associated with a 67% higher risk of hypertension, a 108% higher risk of DM, and a 137% higher risk of metabolic syndrome.^[1]^ In contrast to the diagnosis of nonalcoholic fatty liver disease with a threshold of 5% hepatic fat, there is no generally agreed threshold for the definition of increased fat content in the pancreas. Few studies have focused on the pancreatic fat deposition from the general population.^[7–9]^

Until now, histopathology has been the gold standard for the assessment of pancreatic fat content. However, biopsy of the pancreas is impractical and unethical due to the risk of complications related to its deep retroperitoneal location. It is also important to underline that biopsy measurements are limited by the sampling error.^[10]^

Magnetic resonance spectroscopy (MRS) is currently considered the most sensitive and accurate quantitative measurement of organ fat. However, it requires a high degree of uniformity of magnetic field and shimming is too time consuming. Additionally, spectral imaging may be affected by contamination from visceral fat. This issue arises because the region of interest can be displaced by breathing or diaphragm movement, which may inadvertently include adjacent visceral fat. So, it is usually not recommended for routine clinical application.^[11]^ Magnetic Resonance Imaging (MRI) mDIXON-Quant is a chemical shift-based technique developed in recent years that can accurately quantify the proton density fat fraction (PDFF), and which is now widely used for the noninvasive measurement of tissue fat content.^[12–14]^ PDFF measurements improve the reliability of fat quantification by correcting for confounders such as T2* decay, T1 bias, and noise bias, and for the multispectral complexity of fat.^[15]^ It has the advantages of being noninvasive, rapid, and one-time imaging. Recent studies have shown that it has good consistency with donor studies, biopsy, and ^1^H-MRS^[14,16,17]^; and overcomes the disadvantages of MRS with good repeatability.^[18,19]^ Meanwhile, as shown in our previously study,^[20]^ quantitative CT can also be used to measure the fat content of the pancreas and shows a statistically significant correlation with CSE-MRI measurements of pancreatic fat content.

The aims of this study were: to quantity PDFF of the pancreas in the general population using 3-point Dixon imaging; to explore the changing patterns of the pancreatic fat content of the general population with age; to investigate the relationship between pancreatic fat content and age, gender, and body mass index (BMI).

## 2. Materials and methods

### 2.1. Subjects

This study was approved by the Ethics Committee of Beijing Jishuitan Hospital. All the subjects enrolled in the cohort were healthy volunteers recruited from the local community. The informed consent was obtained for all participants. Two hundred thirty-seven healthy subjects (107 males and 130 females; with an age range of 21–79 years; mean age of 50 ± 15 years) underwent MR exams after giving informed and written consent to the protocol, which was approved by the local review board. None of the subjects was diagnosed with liver or pancreatic disease or dysfunction. Volunteers were grouped according to their age. Age cohorts and numbers of subjects are listed in Table 1.

**Table 1:** The clinical characteristics of the population and fat percentage of the pancreas measured using CSE-MRI PDFF.

	Total	Male	Female
N	237	107	130
Age (yr)	53 (34–63)	45 (34–61)	56.5 (35–63)
Height (cm)	163 (158–170)	171 (167–175)	158 (156–162)
Weight (kg)	68 (60–78)	76 (69.9–83)	62.3 (57.2–68.1)
BMI (kg/m^2^)	25.83 ± 3.64	26.38 ± 3.49	25.38 ± 3.72
Waist (cm)	87 (80–94)	92 (86–98.1)	83 (77–90.2)
Hipline (cm)	98 (94–104)	100 (96–104)	96 (93–104)
Pancreatic fat content (%)	3.33 (1.82–6.16)	3.61 (1.90–7.27)	3.08 (1.76–5.38)

Descriptive characteristics of the study group which are consistent with the normal distribution are presented as mean ± SD. Otherwise, they are presented as the median and inter quartile range.

BMI = body mass index, cm = centimetre, kg = kilogram, N = number of volunteers, m = metre, MRI = magnetic resonance imagimg, PDFF = proton density fat fraction, SD = standard deviation, yr = years.

### 2.2. MR imaging protocol

All subjects underwent a 3-point Dixon sequence using a 3 Tesla MRI (Ingenia, Philips Healthcare, Best, Netherlands) on the upper abdomen. The parameters are as described in our previously published study.^[20]^

**Figure 1.**
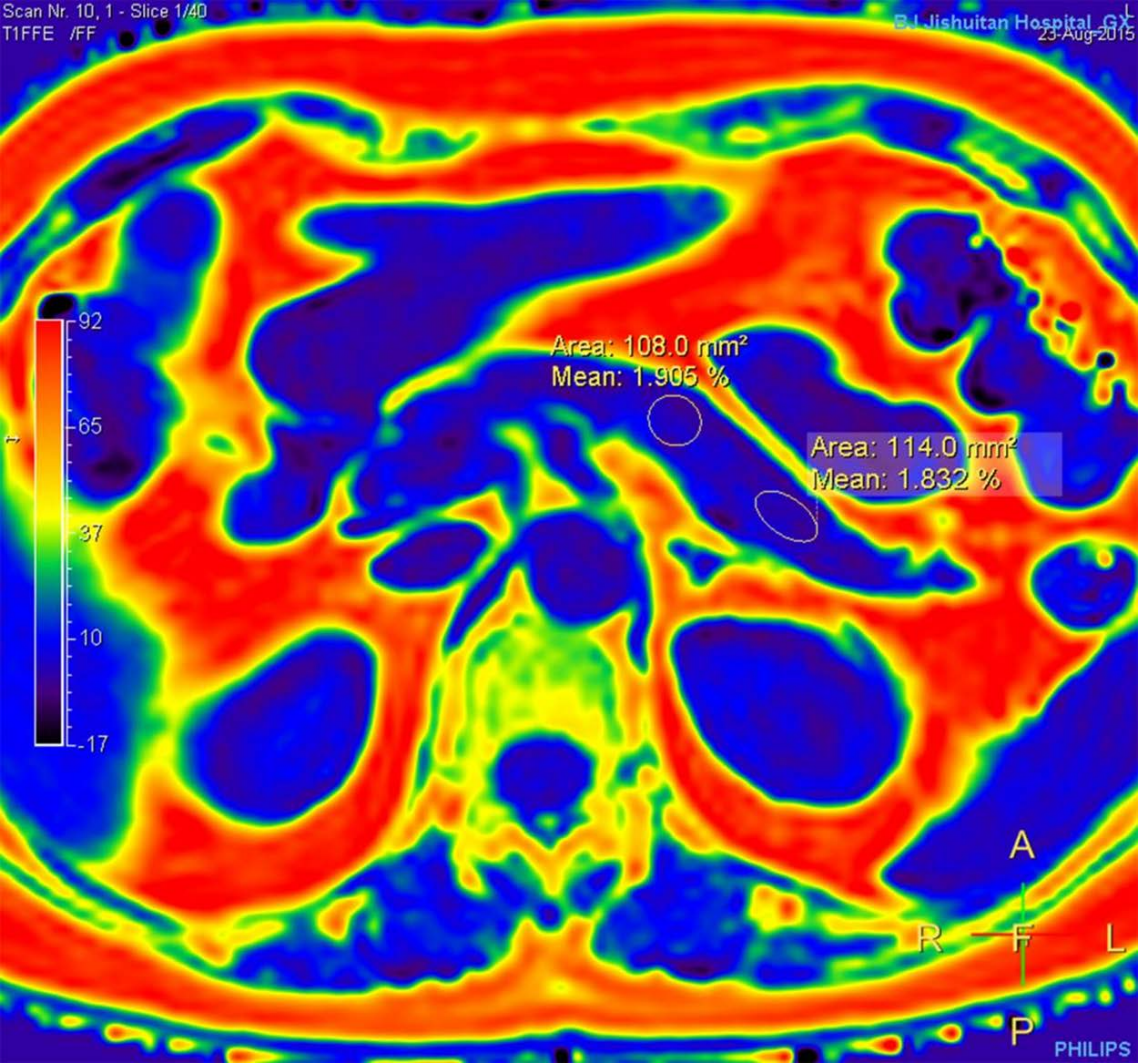
PDFF map shows that ROIs were placed in the body and tail of pancreas. PDFF = proton density fat fraction, ROIs = regions of interest.

After scan acquisition, all data were transmitted to an ISP V7 workstation (Philips Healthcare, Best, Netherlands). Then, 2 experienced radiologists independently measured pancreatic fat content (measured as the PDFF). To measure the PDFF of the pancreas, we use our previously published method^[20]^: circular regions of interest (ROIs) covering an area of approximately 100–130 mm^2^ were manually placed in the pancreatic head, body, and tail where the fat level was homogenous, with care to avoid intrapancreatic vessels and pancreatic ducts (Fig. 1). ROIs were kept small to minimize contamination from extra-pancreatic adipose tissue, making sure that the ROIs were surrounded by pancreatic tissue not only within the imaging plane but also on the slice above and slice below. The mean of all ROIs in each part was calculated to determine the average fat fractions in the head, body, and tail. Then, the mean of all ROIs in the entire pancreas determined the overall pancreatic fat fraction.

### 2.3. Statistical analysis

The fat content of the pancreatic head, body, and tail within the groups was investigated by Friedman analysis of variance test. The height, weight, BMI, waist circumference, hip circumference, and pancreas fat content of the subjects were examined for normality and homogeneity. Descriptive characteristics of the study group that are consistent with the normal distribution are presented as mean ± SD. Otherwise, they are presented as the median. For those groups with normal distribution and homogeneity of variance, one-way ANOVA was used to compare the differences between different age groups of men and women, and an LSD *t* test was used for pairwise comparison. For those who do not conform to normal distribution or have homogeneity of variance, the Kruskal–Wallis H-test was used to compare the differences between different age groups of men and women, and the Nemenyi test was used for pairwise comparison. For the pancreatic fat content data with a normal distribution and equal variance, the differences between males and females in the same age group were compared using the *t* test for 2 independent samples. For data that was not normally distributed, the differences between men and women in the same age group were compared using the Wilcoxon rank sum test for 2 independent samples. Pearson correlation analysis was performed between the pancreatic fat content and age. The relationship between fat content, age, gender, and BMI was analyzed by multiple linear regression. All statistical analysis was performed using SPSS 20.0 (SPSS Inc., Chicago) software. *P* < .05 were considered statistically significant.

## 3. Results

The intraclass correlation coefficient within the 2 observers was 0.997 and 0.996, respectively, and the intraclass correlation coefficient between the 2 observers was 0.989, indicating that the reliability of MRI mDIXON-Quant technique in measuring PDFF is high.

A total of 237 volunteers were included in this study. The clinical characteristics (including gender, age, height, weight, BMI, waist, hipline) and pancreatic fat content of the subjects were statistically described, as shown in Table 1. They are grouped by age and sex. Different genders are divided into 5 groups according to age, as shown in Table 2. The median pancreatic fat content was 3.33% (inter quartile range 1.82–6.16%) and did not vary significantly between the head, body, and tail.

**Table 2:** Pancreatic fat content in different age group and different gender.

Age (yr)	Male		Female	
	N	Pancreatic fat content (%)	N	Pancreatic fat content (%)
21–30	12	3.22	21	1.44de
31–40	32	2.55e	15	1.88de
41–50	16	3.17	16	2.28de
51–60	18	5.23	35	4.41abc
> 60	29	6.26b	43	4.41abc
*H*_*C*_		14.28		33.01
*P* value		.006		< .000

Group 1: 21–30 years old; Group 2: 31–40 years old; Group 3: 41–50 years old; Group 4: 51–60 years old; Group 5: 61–79 years old. a versus group 1; b versus group 2; c versus group 3; d versus group 4; e versus group 5.

N = number of volunteers, yr = years.

A *P* value of <.05 were considered indicative of statistical significance.

**Figure 2.**
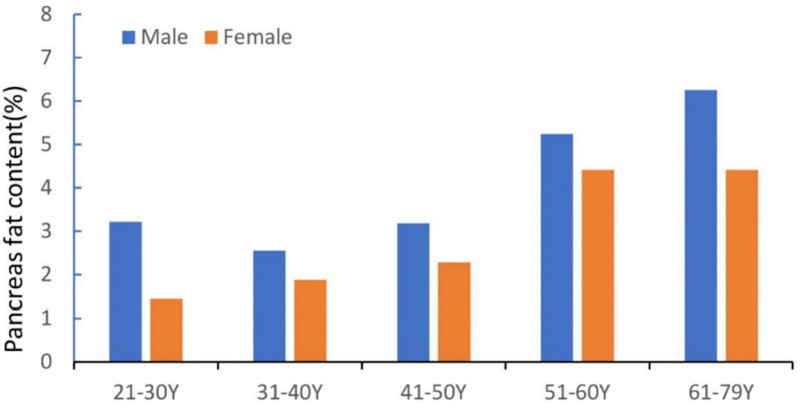
Histogram of pancreatic fat content in 237 subjects from the general population according to age group and gender. Y = years.

Figure 2 shows the distribution of the pancreatic fat content for the different age groups. The ages ranged from 21 to 79 years according to gender. The pancreatic fat content of men and women increased with age.

In men, the increase in PDFF compared with the youngest age group was −20.9%, −1.3%, 62.6%, and 94.4% for groups 2 to 5, respectively. The mean PDFF (6.3%) of the men aged 61 to 79 years was approximately twice that (2.5%) of those aged 31 to 40 years (*P* < .05). In women, the corresponding increases were 31%, 58%, 206%, and 206% compared with the youngest age group. The mean PDFF of women in the 2 oldest groups was 4.4%, which was approximately double that of those aged 21 to 30 years (1.4%), aged 31 to 40 years (1.9%), and aged 41 to 50 years (2.3%) (*P* < .05) (Table 2). There was a positive correlation between pancreatic fat content and age in both men (*r* = 0.336, *P* < .05) and women (*r* = 0.433, *P* < .05).

We used a logistic regression model to assess the association between pancreatic fat and the demographic factors (age, gender, and BMI). The multiple linear regression equation constructed with pancreatic fat content as the dependent variable and the other indicators as the independent variables is: pancreatic fat content = −6.4502–2.171 gender + 0.144 age + 0.314 BMI. Gender negatively affected the content of pancreatic fat. Gender increased or decreased by 1 grade, and the content of pancreatic fat decreased or increased by 2.171%. Age had a positive effect on pancreatic fat content. With the increase or decrease of age by 1 year, pancreatic fat content increased or decreased by 0.144%; BMI had a positive effect on pancreatic fat content, BMI increased or decreased by 1%, and pancreatic fat content increased or decreased by 0.314%.The results showed that the pancreatic fat content was significantly associated with all 3 demographic variables (Table 3). The *P* values of gender, age and BMI were .010, .000 and .090, respectively, which were all < .05, indicating that the difference was statistically significant. The PDFF was higher in men compared with women, and was positively associated with both age and BMI.

**Table 3:** Multiple linear regression analysis between pancreatic fat content (dependent variable) and gender, age, and BMI (independent variables).

Variable	*B*	*t*	*P* -value	95% CI	
				Lowerbound	Upperbound
Constant term	−6.450	−1.913	.057	−13.093	0.193
Sex	−2.171	−2.591	.010	−3.821	−0.520
Age	0.144	5.222	.000	0.090	0.198
BMI	0.314	2.762	.006	0.090	0.538

BMI = body mass index, CI = confidence interval.

*P* < .05 were considered statistically significant.

## 4. Discussion

The mDixon-Quant used in this study is a 6-echo gradient imaging technique that utilizes a 7-peak model and combines T2 * correction to generate FF and T2 * mapping maps. It is a multi gradient, multi echo Dixon technique that has the following advantages^[15,21]^: By imaging, 6 echoes can be collected at once, generating 6 sets of images. Water and fat signals are separated in positive and negative phases, and the 7-peak fat model is used to generate 6 sets of images, including positive phase, negative phase, water phase, and fat phase T2*、R2*. In addition, T2 * correction can accurately quantify fat content; Short imaging time, only 12.5 seconds, more suitable for elderly patients with poor breath holding, can be scanned with just 1 breath hold; The post-processing is simple and can directly provide the percentage of fat content; The fat content of the entire pancreas can be measured arbitrarily and repeatedly, with a wide range of measurable fat ratios (0–100%). Tissue with fat content > 50% (such as bone marrow, subcutaneous, and visceral fat) can also provide measurement results; No radiation damage, simple operation, suitable for physical examination screening and multiple clinical follow-up examinations, and relatively more suitable for fat quantification of small organs compared to MRS.^[21]^

The ectopic deposition of visceral fat, especially in the pancreas, is a significant factor in the pathogenesis of diabetes and metabolic syndrome. Previous studies have focused on the fat content in the pancreas and its possible associations with type 2 diabetes and metabolic syndrome.^[6,13,22]^ However, achieving a better understanding of the clinical significance of pancreatic fat deposition requires comparison with data acquired from a normal population. It is therefore important to confirm the threshold of normal pancreatic fat content and establish how the fat content varies with age and gender.

In our study of a population of healthy individuals of both sexes over a wide age range, the median pancreatic fat content was 3.33% (inter quartile range 1.82–6.16) over all subjects. This result is consistent with that of Kuhn et al^[8]^ who reported a pancreatic fat content of 4.4% in the general population. In another study, Ringh et al^[1]^ had suggested 6.2% as the threshold for normal pancreatic fat content in imaging studies. However, the studies in this systematic review used different quantitative approaches to MRI fat measurements and included individuals with a limited range of BMI (on average, below 30 kg/m^2^). In contrast, all the subjects enrolled in our study had their pancreatic fat content measured using the same method, and the study included individuals with a BMI higher than 30 kg/m^2^. Hence, their threshold of 6.2% cannot be compared directly with our result.

In agreement with previous studies,^[7,23]^ our study demonstrated that the prevalence of pancreatic fat in the general population increases with age. It is assumed that the pancreas degrades and that the incidence of pancreatic steatosis increases with increasing age. Consistent with the findings of Li et al^[7]^, our results showed that pancreatic fat deposition in men increases above the age of 50 and that PDFF in the oldest group (61–79 years) was approximately twice that in the 3 youngest groups (21–50 years). In contrast to the study of Li et al^[7]^, both sexes were recruited in our study, and we also assessed the relationship between age and pancreatic fat content in female volunteers. Our result showed that there was a positive correlation between pancreatic fat content and age in women as well as men. The PDFF in women aged 51 to 79 was approximately twice that of those aged 21 to 50 years.

The present study also provides more detailed insights into the association between gender and pancreatic fat content. PDFF in men was higher than that in women in the same age group. However, women showed a greater increase in pancreatic fat content with age compared with men, which is different from the finding of Kuhn et al.^[8]^

The relevance of BMI to pancreatic fat content is currently under debate. In our study, PDFF of the pancreas was positively associated with BMI, in agreement with most previous studies.^[8,24–26]^ It is well known that when BMI increases, the amount of visceral fat also increases. A high BMI predisposes to metabolic syndrome, while metabolic syndrome is itself associated with pancreatic fat content. Despite this, Patel et al^[22]^ reported that the pancreatic fat content had no correlation with BMI. We hypothesized that the reason might be the inclusion in their study of patients with nonalcoholic fatty liver disease. In contrast, all the subjects in our study were healthy volunteers.

It is well known that the normal pancreas has an irregular boundary and that it atrophies with age. The homogeneity of fat distribution within the pancreas has been debated for many years.^[7,8,22,27–31]^ Our result shows that the PDFF does not vary significantly between the head, body, and tail, in agreement with most previous studies.^[7,8]^ However, some studies have indicated that the fat tends to be deposited in the anterior part of the head of the pancreas, instead of the back of the head and the uncinate process.^[30,32,33]^ In our measurements, we found that the mean PDFF of larger ROIs was higher than that of smaller ROIs. This implies there may be visceral fat infiltrating the boundary of the pancreas. Thus, it is possible that any inter-lobular intrusion of visceral fat might be interpreted as intrapancreatic fat and hence overestimate the results of the study. Therefore, compared to a previously published study,^[31]^ we chose a small ROI (approximately 100–130 mm^2^) to minimize contamination from volume averaging with extra-pancreatic adipose tissue, making sure that the ROIs were surrounded by pancreatic tissue not only within the imaging plane, but also on the slice above and slice below. The result showed PDFF did not vary significantly between head, body, and tail, in agreement with most previous studies. In addition, Al-Mrabeh et al presented a new method to minimize the extent of inclusion of extrinsic tissues, and the interobserver variation was substantially reduced when pancreas fat levels were moderately high.^[34]^

There are several limitations in the current study. First, the 3-point Dixon technique has been proved in previous studies,^[12–14,16,17]^ but we didn’t compare it with histologically measured steatosis to verify the accuracy in our study. Second, the general population recruited in our study included both normal and overweight individuals, but none with severe obesity. Additionally, patients with relevant diseases such as DM or cardiovascular were not included in the study. Future studies will focus on the pancreatic fat content of severely obese subjects.

In conclusion, pancreatic fat was found to be homogeneous and evenly distributed in the head, body, and tail of the pancreas. The PDFF of the pancreas is correlated with age, gender, and BMI. It is higher in elderly people, in obesity, and men. However, women showed a more rapid increase in the deposition rate of fat with age than men. The increase in pancreatic fat deposition accelerates significantly from the sixth decade in men and from the fifth decade in women.

## Author contributions

**Data curation:** Xiaoguang Cheng.

**Investigation:** Wenjun Yao.

**Methodology:** Wenjun Yao.

**Writing** – **original draft:** Xue Qi, Fang Zhang.

**Writing** – **review & editing:** Liming Guan.
